# Risk Factors and Clinicopathological Features for Developing a Subsequent Primary Cutaneous Squamous and Basal Cell Carcinomas

**DOI:** 10.3390/cancers14133069

**Published:** 2022-06-23

**Authors:** Magdalena Ciążyńska, Marta Pabianek, Martyna Sławińska, Adam Reich, Bogumił Lewandowski, Katarzyna Szczepaniak, Małgorzata Ułańska, Dariusz Nejc, Robert Brodowski, Michał Sobjanek, Witold Owczarek, Grażyna Kamińska-Winciorek, Dariusz Lange, Monika Słowińska, Katarzyna Wróbel, Andrzej Bieniek, Anna Woźniacka, Anika Pękala, Łukasz Kuncman, Magdalena Salińska, Marcin Noweta, Małgorzata Skibińska, Joanna Narbutt, Karol Ciążyński, Marta Lewandowska, Elżbieta Dziankowska-Zaborszczyk, Aleksandra Lesiak

**Affiliations:** 1 Department of Proliferative Diseases, Nicolaus Copernicus Multidisciplinary Centre for Oncology and Traumatology, ul. Pabianicka 62, 93-513 Lodz, Poland; pabianek.martaaa@wp.pl (M.P.); katarzynaa.szczepaniak@wp.pl (K.S.); malgo.ulanska@wp.pl (M.U.); anikapekalka@op.pl (A.P.); 2 Department of Dermatology, Paediatric Dermatology and Oncology Clinic, Medical University of Lodz, 91-347 Lodz, Poland; marcinnk.nowveta@wp.pl (M.N.); skibka.malgo@wp.pl (M.S.); joanka.narbu@wp.pl (J.N.); leska.leska.ola@wp.pl (A.L.); 3 Department of Dermatology, Venereology and Allergology, Medical University of Gdansk, 80-214 Gdansk, Poland; martynka.slawinska@wp.pl (M.S.); michalko.sobjanek@wp.pl (M.S.); 4 Department of Dermatology, University of Rzeszow, 35-310 Rzeszow, Poland; adamo.reicho@wp.pl; 5 Clinical Department of Maxillo-Facial Surgery, Frederic Chopin Provincial Specialist Hospital, 35-310 Rzeszow, Poland; bogumilo.lewandowski@wp.pl (B.L.); roberto.brodowski@wp.pl (R.B.); 6 Department of Surgical Oncology, Medical University in Lodz, Nicolaus Copernicus Multidisciplinary Centre for Oncology and Traumatology, 93-513 Lodz, Poland; daiuszz.nejca@wp.pl; 7 Dermatology Clinic, Military Institute of Medicine in Warsaw, 04-141 Warsaw, Poland; owczarko.witko@wp.pl (W.O.); slowinska.monika2@wp.pl (M.S.); katrzyn.wrobko@wp.pl (K.W.); 8 Department of Bone Marrow Transplantation and Hematology-Oncology, The Maria Skłodowska-Curie Memorial Cancer Centre and Institute of Oncology, Branch in Gliwice, 44-102 Gliwice, Poland; grazyna.kamin.winciorek@wp.pl; 9 Department of Tumor Pathology, The Maria Skłodowska-Curie Memorial Cancer Centre and Institute of Oncology, Branch in Gliwice, 44-102 Gliwice, Poland; darioszk.langg@wp.pl; 10 Bieniek Medical Center, 52-016 Wrocław, Poland; bienko.andrewo@wp.pl; 11 Department of Dermatology and Venereology, Medical University of Lodz, 90-419 Lodz, Poland; ana.wozniacka2@wp.pl (A.W.); magdal.salk@wp.pl (M.S.); 12 Department of Radiotherapy, Medical University of Lodz, 93-513 Lodz, Poland; lukss.konc@wp.pl; 13 Institute of Applied Computer Science, Lodz University of Technology, 90-537 Lodz, Poland; karol.ciazynski@gmail.com; 14 Department of Infectious Diseases and Hepatology for Adults, Medical University of Lodz, 93-513 Lodz, Poland; marta.lskaws@wp.pl; 15 Department of Epidemiology and Biostatistics, Medical University of Lodz, 90-419 Lodz, Poland; elk.dziank.zabor@wp.pl

**Keywords:** non-melanoma skin cancer, keratinocyte carcinomas, basal cell carcinoma, squamous cell carcinoma, non-invasive diagnosis and monitoring

## Abstract

**Simple Summary:**

Patients with basal cell carcinoma (BCC) and cutaneous squamous cell carcinoma (SCC) often develop new keratinocyte carcinoma (KC), but information is limited on the frequency and timing of these subsequent tumors. This information is crucial to guide follow-up care. Given the significant clinical differences of the characteristic feature of individual skin cancer, estimation of the risk of a subsequent tumor should be estimating separately. The aim of our retrospective study was to assess risk factors for a subsequent skin cancer development. We demonstrated that patients with multiple tumors must be followed up carefully and for a long time. Moreover, we indicated the connection between the BCC subtype and increased risk for further KC development. BCC subtypes with an aggressive growth pattern predispose not only to increased risk for the recurrence but also are expected to be at an increased risk for a subsequent tumor. The non-invasive diagnosis, monitoring and follow up should be more comprehensive for those patients compared to low-risk BCC.

**Abstract:**

Background: Patients with diagnosed keratinocyte carcinomas (KCs) have an increased risk of subsequent skin cancers development. Current studies indicate that patients with subsequent tumors should be followed up regularly. However, none of the studies indicate the connection between the specific subtypes and an increased risk for further KCs development. The study assesses the differences in the risk of developing a subsequent skin cancer after a previous diagnosis of KC, especially considering individual types of skin malignances, and identifies potential factors associated with an increased risk of new cutaneous tumor describing non-invasive diagnosis and monitoring. Methods: Pathology and medical records were examined to identify the characteristics of patients with multiple KCs diagnosed between 1999 and 2019. Results: The study group comprised 13,913 KCs occurring in 10,083 patients. Multiple KCs were observed in 2300 patients (22.8%). The analysis showed aggressive subtypes, multiple tumors, and male sex as significant prognostic factors. Conclusions: The most crucial risk factors for developing subsequent KC are being of a male gender, an aggressive tumor subtype, and previous history of multiple skin cancers. Basal cell carcinoma subtypes, such as infiltrative basosquamous, with aggressive growth patterns predispose not only to increased risk for the recurrence but are also expected to be at higher risk of subsequent KCs.

## 1. Introduction

The skin is the largest human organ, which constitutes the first and the most external barrier protecting organism against invasion from pathogens and stress agents, such as ultraviolet radiation UVR [1,2], which is considered as the most significant factor in the growth of most skin cancers. Significant world-wide increase in the incidence of cutaneous neoplasm has been observed in recent years. It is widely recognized that 20% of people will develop a skin tumor in their lifetime [3]. Cutaneous neoplasms usually include melanomas and non-homogenous keratinocyte carcinomas (KCs) based on the cell of origin and clinical behavior. KCs include basal (BCC) and squamous (SCC) cell carcinomas derived from epidermal keratinocytes. Recent data indicates that BCC representing 80% of all KCs is not a homogeneous disease and its specific histological subtypes that present various clinical characteristics are located at specific body locations and may present various etiology, recurrence risks after standard excision, as well as a tendency to develop secondary skin neoplasm generation [4]. Subtyping of BCC as a prognostic factor is a well-established idea [5]. These subtypes, as indicated by The National Comprehensive Cancer Network (NCCN) guidelines, are defined as an “aggressive growth pattern,”, including infiltrative, basosquamous (BSC), and morpheaform, and are more likely to recur than the superficial and nodular BCC [6,7,8,9].

The phenomena of the occurrence of multiple primary cancers within one organ, especially skin was already documented [10,11,12]. Recent data indicate that patients with first time diagnosed skin cancer have an elevated risk of the subsequent cutaneous neoplasms developing [13,14,15]. Most published studies on the risk of secondary skin cancer consider BCC and SCC together, as KC. Individual BCC subtypes are not analyzed separately. However, the risk factors of a subsequent skin cancer might differ for each skin cancer subtype.

The early detection of subsequent KC is crucial since it usually is associated with a smaller tumor size, which is connected with a reduced scar and the improvement of the cosmetic result, as well as a decrease in the cost of therapy linked with repeat surgery. Additionally, for BSC, and especially for SCC, reducing the possibility of metastasis disease development and even avoiding mortality makes the early detection of this cancer an even better advantage.

Therefore, the aim of the study is to assess the differences in the risk of developing a subsequent skin cancer after the diagnosis of a cutaneous neoplasm, especially considering individual types of skin malignances, and to recognize potential features linked with an amplified risk of new cutaneous tumor.

## 2. Materials and Methods

We retrospectively searched histopathological databases for records between 1999 and 2019 in multiple tertiary dermatology and oncology centers in Poland, including Rzeszow, Warsaw, Lodz (two sites), Gdansk, Wroclaw, and Gliwice. All patients aged 18 years old and above with histopathologically diagnosed KC were included in the research. The patients with basal cell nevus syndrome, a genetic condition predisposing to development of multiple BCCs, were excluded for the study. All the patients were Caucasian with skin phototypes ranged between I and III according to Fitzpatrick scale (I: never tan always burn, II: sometimes tan often burn, III: often tan sometimes burn) [16]. Both private hospitals and national health centers were included in the study. The patient’s recorded data included age and sex, site of the cancer, number of primary skin tumors, timing between tumor occurrences, size of a tumor, and histopathological diagnosis. Recurrent skin cancers were identified and excluded from analysis based on the data from previous diagnosis. A recurrent and a new BCC was distinguished based on histopathological and clinical data. The recurrence referred to a skin tumor that reappeared at a site after an attempt to remove it by surgery or some other means. The second primary skin cancer is defined as a tumor found at a different site and/or after a delay in time after the first primary skin cancer. Thus, tertiary (third, fourth, fifth, etc.) primary skin cancer is defined as a tumor located at different site and time compared to all previous primary skin cancers.

Study was approved by Human Research Ethics Committee of the Medical University of Lodz, Poland (RNN/209/18/KE). The study was conducted according to Declaration of Helsinki. to All organizations the subjects’ data were being sourced from provided the informed consent. The Statistica 13.0 was used to statistically analyze the results. The Student’s *t*-test, analysis of variance (ANOVA) post-hoc comparisons, χ^2^ test, and Fisher’s exact test were used for data calculation. *p* values less than 0.01 were considered as statistically significant. The univariable and multivariable analysis was performed and presented using hazard rations (HR) and 95% confidence intervals (CI).

## 3. Results

Skin cancer characteristics were analyzed with specific focus on the number of cutaneous neoplasms diagnosed, the timing of the first and subsequent tumor, as well as the location and the prevalence of subtypes skin cancer. Moreover, these features among patients with each type of multiple KCs were compared to those of patients with single skin cancer. Table 1 presents the clinical variables of the patients with single and multiple KCs.

A total of 10,086 patients were diagnosed with at least one excised and histologically confirmed KC. The multiple primary KC was observed for 2300 patients (22.8%). Of these patients, 68% had two primary tumors, 17% had three primary tumors, 7% had four, 3% had five, 2% had six, and 3% had seven or more.

There were 3581 men and 4205 women with single KC, while there were 1224 men and 1076 women with multiple KC. The male predominance has been observed for multiple KCs (*p* < 0.01). The mean age of the patients with single KC was 70.16 ± 10.2 years and that of the patients with multiple KCs was 71.17 ± 11.4 years (*p* = 0.15). 

The anatomical distribution of the KCs was collected in 10,778 cases (7213 single KC cases and 3565 multiple KC cases). In both groups, KC lesions were located mainly on the face (77.97% for single KC and 74.33% for multiple KC). The second most frequent site was the trunk, followed by lower limb, upper limb, neck, and scalp. No significant differences have been observed regarding the anatomical distribution and predisposition to the development of multiple KCs.

We performed Cox proportional hazard regression analysis to estimate variables at first assessment potentially associated with the development of new skin neoplasm. The multivariate analyses, the diagnosis of a high-risk BCC (adjusted HR = 2.01; 95% CI = 1.73–2.23; *p* < 0.0001), the history of KC development (HR = 1.91; 95% CI = 1.63–2.21; *p* < 0.0001) and male gender (HR = 1.37; 95% CI = 1.25–1.51; *p* < 0.0001) were significantly associated with the development of new skin neoplasm (Table 2). For patients with multiple KC the distribution of the sites of subsequent KCs was similar to that of the initial KCs. The initial and second KC occurred on the same body site in 76.9% of patients (1595/2073). Location of the first and second primary KCs is presented in Table 3. Of the 292 patients who had an initial KC on the trunk region, 115 (39.4%) had a second primary tumor at this anatomical site. Of the 79 patients who had an initial primary tumor on the upper limb, 30 (38.0%) also had a second KC on the upper limb. The highest body site concordance was seen among the 1613 patients who had an initial primary KC on the face; 1372 (85.1%) of these patients had a second primary KC on the face.

Within all KC cases we observed 11,848 BCCs and 2065 SCCs. Both for single and multiple KCs the BCC subtype was predominant (single KCs: 85.00%, multiple KCs: 83.26%). No significant differences have been observed regarding the BCC or SCC type and predisposition to multiple KC development (Table 1). Multiple KC occurred in 25% for the first SCC, while the subsequent KC occurred in 22% for the first BCC. 

Information concerning the subtype of BCC was recorded in 5479 cases (3655 single KC cases and 1824 multiple KC cases). The nodular BCC was the most common subtype of BCC for both single and multiple KCs (51.74% for single KC; 39.12% for multiple KC), followed by superficial (31.71% for single KC; 32.20% for multiple KC), infiltrative (13.38% for a single KC; 23.52% for multiple KC), and basosquamous (3.17% for a single KC; 5.16% for multiple KC). High-risk BCC (infiltrative and basosquamous) presents statistically significant predominance for multiple BCC development in comparison to low-risk BCC (nodular and superficial) (*p* < 0.01).

For patients with multiple KC, the type of subsequent KCs was similar to that of the initial KCs (Table 4). For cases where the first lesion was BCC in 93.7%, the second primary KC was also BCC, while where the first primary KC was SCC, the second was also SCC in 66.4%. For patients with multiple KC, the specific BCC subtype of subsequent KCs was similar to that of initial KCs. The initial and second primary KC occurred with the same BCC subtype in 61.3% of patients (209/341). Moreover, the third, fourth and fifth primary KC occurred at a similar location as the first primary lesion (Table 5).

**Figure 1:**
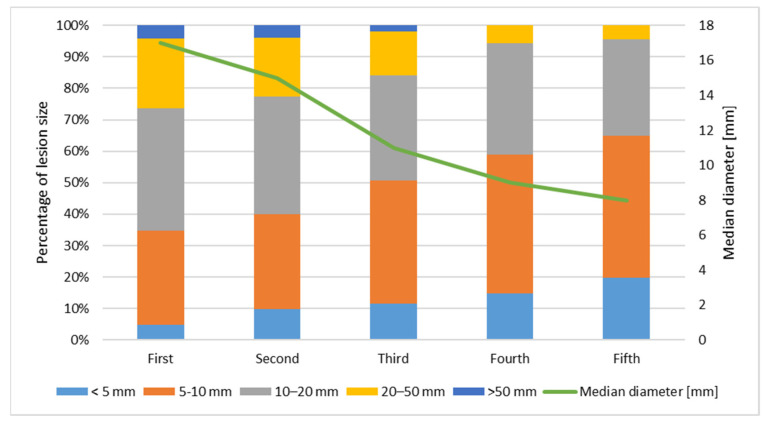
Size of subsequent primary KC.

**Figure 2:**
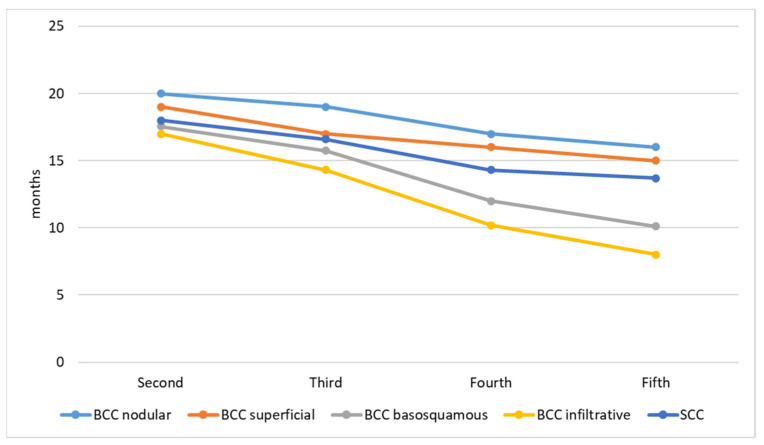
Timing between subsequent primary KC development.

The size of the primary KC decreases with the number of lesions. The first primary KC has the biggest diameter, while the second, third, fourth, and fifth decreases, respectively. Figure 1 presents the size of lesions. The timing of subsequent primary KC development is presented in Figure 2. The time interval between the first and second lesion diagnosis is higher in comparison to the time between the second and third lesion, and further decreases for the next subsequent lesion development. One KC put patients at risk of a second primary KC, and a second primary KC put patients at an even higher risk of a third primary KC. Moreover, both infiltrative and basosquamous BCCs tend to develop faster in comparison to nodular and superficial BCCs.

## 4. Discussion

Patients with skin cancer are at a higher risk of other KC development [17,18]. Currently, most studies focus on the analysis of subsequent BCC and SCC cases without an investigation of the specific BCC subtypes [13]. In this study, the risk of developing a subsequent SCC and BCC in patients with former skin cancer was analyzed taking into consideration the certain subtype of BCC to provide a comprehensive view on the current data. 

Multiple primary KCs were recognized in 22.8% of a constructed cohort of patients from our databases, which is consistent with various studies, and the incidence ranged between 20% and 67% [19,20,21,22,23,24,25,26,27,28]. However, the risk of a subsequent KC developing differs depending on the primary subtype. Patients diagnosed with infiltrative or basosquamous BCC are about two times more likely to grow another KC than patients diagnosed with less aggressive BCC subtypes, such as nodular and superficial. The higher risk for subsequent skin cancer development was not observed for patients with primary SCC and were similar to patients with nodular and superficial BCC. These discoveries are outstanding as it may be anticipated that patients developing primary SCC could tend to an increase in probability of further KCs, as SCC is defined as a more aggressive KC type.

Males were at higher risk of further KC development, which is in accordance with previous research [24,25,28,29]. It is unexpected that males may be genetically more predisposed to develop multiple KCs, but this requires further studies. Probably, females are more eager to adjust their attitude and behavior after the diagnosis of a first skin cancer to protect from a subsequent cancer, while males often do not. These behavioral changes are confirmed by other studies. Chen et al. [30] investigates gender-based differences in skin protection behaviors after skin cancer treatment. Their results showed that a larger percentage of females compared to males adopted behavioral changes to prevent future skin cancers. Moreover, females were more likely to self-examine their skin for abnormal markings. Possibly, for the same reason females are also younger at the time of the first diagnosis [31]. Patients who develop a single KC are at a similar age to patients who develop subsequent skin cancer. Thus, the risk for multiple tumor development, was not associated with higher age. 

The anatomical location of tumors is not a risk factor of new tumor development [29]. However, in most cases (76.9%) they had their subsequent primary skin cancer at a similar body location as the first primary lesion. The relationship between ultraviolet radiation exposure and subsequent KC development remains unclear. We observed that patients having their first primary KC located at sun-exposed areas, such as face, also tend to develop subsequent skin cancer at the same location. The similar observation was not noticed for patients having their first skin cancer at sun protected areas, such as trunk, since the second location of the lesion was either the trunk or another body site, which could be associated with individual genetic predisposition. This should be considered when searching for the occurrence of subsequent tumors and makes comprehensive physical examination advisable. It is known that KC development is the result of cumulative life exposure to UV radiation. However, some studies have found a different etiology [32,33], presenting that sun exposure in childhood may be important in the development of KC, also increasing the risk for subsequent KCs.

We observed the relationship between the subtype of the first primary KCs and the type of the second one. Patients with history of a BCC are eight times more likely to develop another BCC than a first SCC. Contrastingly, the patients already diagnosed with a SCC are two times more likely to develop consecutive SCCs than BCC.

The higher risk of subsequent BCC or SCC development after a first KC indicates a partially common etiology of ultraviolet-induced field cancerization, as well as a genetic predisposition to this skin cancer [34,35]. People were most likely to develop the same subtype of skin cancer, and this suggests that there are strong differences in associated risk factors and carcinogenesis among the KCs. 

**Figure 3:**
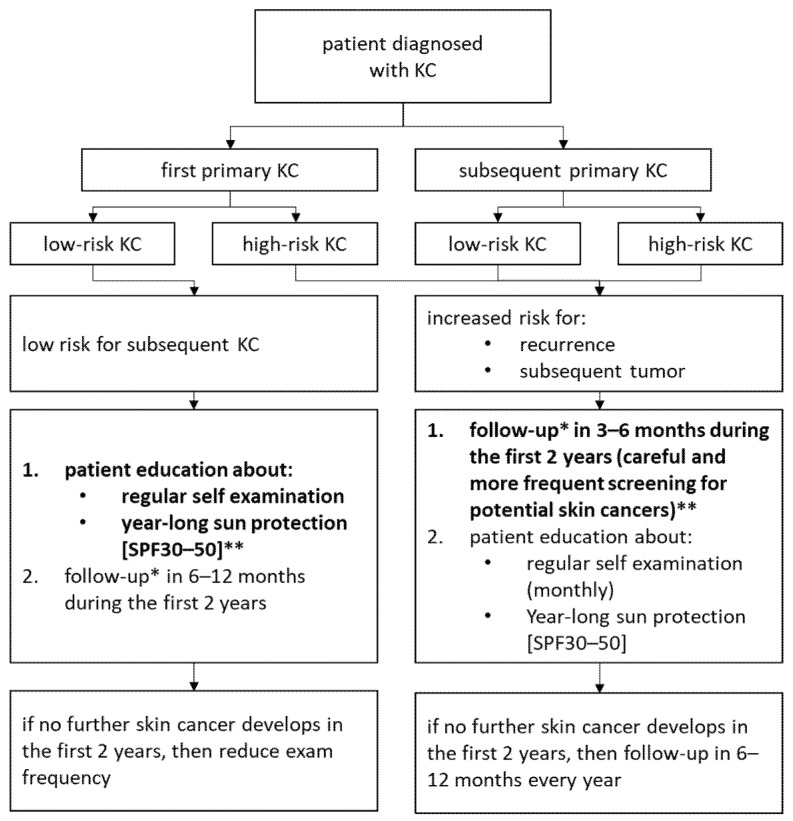
Recommendation for follow up depending on skin cancer. * follow up: full body dermatology exam and dermatoscopy. ** the crucial prevention marked in bold.

In patients with a history of infiltrative BCC it is highly probable that the second skin malignancy is also infiltrative BCC. Similar observation has been noticed for another aggressive BCC subtype, such as BSC. However, the first occurrence of BCC with lower aggressiveness, such as nodular, tends to develop either nodular or superficial BCC, but with much less frequency in comparison to the more aggressive BCC subtypes. We observed that people not developing a subsequent skin cancer by two years after a first lifetime nodular or superficial BCC diagnosis were at a lower risk for a subsequent tumor. Thus, clinicians could consider if these patients may be visited by the clinic less regularly (Figure 3).

The risk for a second KC development after a first lifetime KC is significantly lower in comparison to the risk for a third KC after a second one. Consequently, the risk increases in proportion to the number of diagnosed skin neoplasms. We found that 25% of the skin cancer patients have their second subsequent primary lesion within the first year, those results are higher than reported by Wehner et al. [18], who documented 14.5% of the second primary tumors within the first year. The time intervals between the subsequent tumor decreases proportionally to the number of diagnosed tumors. Moreover, basosquamous and infiltrative BCC subtypes tend to develop a subsequent KC earlier in comparison to low-risk BCC subtypes.

Based on a tumor size, the biggest skin neoplasm was the first one for most patients. The size of a diagnosed KC also decreased with the next subsequent skin cancer, confirming the tendency toward smaller subsequent cutaneous neoplasm. This tendency toward smaller KCs is probably due to better observation, skin monitoring, and early detection.

Our study has some limitations due to the retrospective character. The study identifies the major risk factors for subsequent primary KCs and presents recommendations for follow up, based on analysis and data available in histopathological databases and medical records. The prospective cohort study could be beneficial to analyze the data regarding family members involved, a history of sunburns, cumulative sun exposure, and a history of immune suppression as well. Wallberg et al. [36] suggested that recorded skin tumors among relatives (siblings or parents) is a strong risk factor associated with a subsequent BCC. Moreover, they found that patients sunburned after the age of 60 had significantly increased risks associated with multiple BCCs. The uniform population could be another limitation for the study since the research focuses on Poland and does not consider genetic susceptibility. Hallaji et al. [37] found that the patients who were born in mountainous area had a significantly increased risk of multiple BCCs. Moreover, they suggested that a history of radiotherapy and abnormal underlying skin at the site of the tumor may be secondary to a history of radiotherapy as the strong risk factors for multiple BCCs. Thus, further studies could focus on the genetic factors associated with predisposition to subsequent skin cancer development, a history of radiotherapy and a family history of skin tumors considering all types of BCCs and SCCs, including the pigmented pathologic type of BCC. 

## 5. Conclusions

It is the first study that evaluates the differences in the risk of developing a subsequent KC depending on the primary diagnosing skin cancer subtype. We have shown that the crucial risk factors for subsequent KC development are male gender, an aggressive tumor subtype, and a previous history of multiple skin cancers. This observation has strong implications for clinical care, non-invasive diagnosis, and monitoring. The risk for the next new KC over time is significantly lower after a first BCC or SCC diagnosis than a previous medical history of multiple skin cancer diagnosis. BCC subtypes with aggressive growth patterns predispose not only to increased risk for the recurrence, but also they are expected to be at an increased risk for a subsequent tumor. Patients with their first KC, especially low aggressive subtypes of skin cancer, might be the group most likely to use preventive counseling, while patients presenting with a previous diagnosis of infiltrative or basosquamous subtypes of BCC might benefit from careful and more frequent screening for potential skin cancers. 

Thus, a prevention is essential in patients with a history of KC, and patients must be counseled about sun protection and the future potential risk of subsequent KCs. All skin cancer patients should not only be recommended a lifetime annual dermatological check-up for subsequent skin cancer, but also perform self-examination and behavior modification.

## Figures and Tables

**Table 1:** Clinical variables of patients with single and multiple KC.

	Single KC		Multiple KC		
**Total Number of Patients**	**7786**		**2300**		***p* value**
**Sex**					*p* < 0.01
Male	3581	45.99%	1224	53.22%	
Female	4205	54.01%	1076	46.78%	
**Age**					*p* = 0.15
Mean	70.16		71.17		
Min	20		29		
Max	102		87		
**Location of first primary KC**					*p* = 0.07
Face	5624	77.97%	1613	74.33%	
Trunk	827	11.47%	292	13.46%	
Upper limb	210	2.91%	79	3.64%	
Lower limb	274	3.80%	82	3.78%	
Scalp	111	1.54%	49	2.26%	
Neck	167	2.32%	55	2.53%	
Unknown	573		130		
**Type of first primary KC**					*p* < 0.01
SCC	1168	15.00%	385	16.74%	
BCC	6618	85.00%	1915	83.26%	
Nodular	1891	51.74%	554	39.12%	
Superficial	1159	31.71%	456	32.20%	
Infiltrative	489	13.38%	333	23.52%	
Basosquamous	116	3.17%	73	5.16%	
Unknown BCC subtype	2963		499		

**Table 2:** Results of the univariable and multivariable Cox proportional hazard regression analyses of all subsequent KC survival. HR, hazard ratio; 95% CI, 95% confidence interval.

	Univariate and Multivariable Analysis: HR (95% CI); *p* Value
Male gender ^a^	1.37 (1.25–1.51); *p* < 0.0001
Diagnosis of a BCC ^b^	0.88 (0.77–1.01); *p* = 0.064
Diagnosis of a high-risk BCC ^c^	2.01 (1.73–2.34); *p* < 0.0001
History of KC development ^d^	1.91 (1.63–2.21); *p* < 0.0001
Age ^e^	0.93 (0.87–9.98); *p* = 0.132
Size of primary lesion ^e^	1.06 (1.01–1.11); *p* = 0.173

^a^ Female gender was defined as reference category. ^b^ Diagnosis of a SCC was a reference category. ^c^ Diagnosis of a low-risk BCC was a reference category. ^d^ Lack of subsequent skin neoplasm was defined as a reference category. ^e^ Univariate analysis.

**Table 3:** Location of non-melanoma skin cancers.

Site of First Primary KC	Site of Second Primary KC														
	Face		Trunk		Upper Limb		Lower Limb		Scalp		Neck		Unknown		Total
Face	1372	85.1%	75	4.6%	23	1.4%	22	1.4%	16	1.0%	32	2.0%	73	4.5%	1613
Trunk	111	38.0%	115	39.4%	26	8.9%	14	4.8%	6	2.1%	5	1.7%	15	5.1%	292
Upper limb	23	29.1%	13	16.5%	30	38.0%	4	5.1%	1	1.3%	4	5.1%	4	5.1%	79
Lower limb	24	29.3%	8	9.8%	7	8.5%	40	48.8%	0	0.0%	0	0.0%	3	3.7%	82
Scalp	21	42.9%	4	8.2%	1	2.0%	3	6.1%	16	32.7%	3	6.1%	1	2.0%	49
Neck	22	40.0%	7	12.7%	0	0.0%	2	3.6%	1	1.8%	22	40.0%	1	1.8%	55
Unknown	67	51.5%	23	17.7%	4	3.1%	5	3.8%	1	0.8%	4	3.1%	26	20.0%	130
Total	1640		245		91		90		41		70		123		2300

Data is marked with grey background when the second lesion occurred in the same location as the first one.

**Table 4:** Type of non-melanoma skin cancers.

Type of First Primary KC		Type of Second Primary KC															
		SCC		BCC		BCC Subtype											Total
						Nodular		Superficial		Infiltrative		Basosquamous		Unknown BCC		Total BCC	
**SCC**		**221**	62.3%	134	37.7%	17	4.8%	5	1.4%	3	0.8%	0	0.0%	109	30.7%		355
**BCC**		243	12.5%	1702	87.5%	567	29.2%	643	33.1%	323	16.6%	66	3.4%	103	5.3%		1945
**BCC subtype**	Nodular	71	12.7%	488	87.3%	289	51.7%	96	17.2%	12	2.1%	7	1.3%	84	15.0%	559	
	Superficial	69	11.4%	535	88.6%	74	12.3%	427	70.7%	16	2.6%	15	2.5%	3	0.5%	604	
	Infiltrative	18	5.5%	312	94.5%	86	26.1%	62	18.8%	155	47.0%	5	1.5%	4	1.2%	330	
	Basosquamous	6	8.1%	68	91.9%	6	8.1%	14	18.9%	17	23.0%	31	41.9%	0	0.0%	74	
	unknown BCC	79	20.9%	299	79.1%	112	29.6%	44	11.6%	123	32.5%	8	2.1%	12	3.2%	378	
**Total**		464		1836													2300

Data is marked with grey background when the second lesion subtype is the same as the first one.

**Table 5:** Pathological characteristics of multiple KC.

	First		Second		Third		Fourth		Fifth	
	*n* =	2300	*n* =	2300	*n* =	736	*n* =	347	*n* =	182
Location of primary KC										
Face	1613	74.3%	1640	75.3%	531	72.1%	236	68.0%	132	72.5%
Trunk	292	13.5%	245	11.3%	93	12.6%	52	15.0%	24	13.2%
Upper limb	79	3.6%	91	4.2%	41	5.6%	23	6.6%	11	6.0%
Lower limb	82	3.8%	90	4.1%	29	3.9%	13	3.7%	2	1.1%
Scalp	49	2.3%	41	1.9%	18	2.4%	10	2.9%	3	1.6%
Neck	55	2.5%	70	3.2%	25	3.4%	13	3.7%	10	5.5%
Unknown	130		123		0		0		0	
Size of primary KC [diameter, mm]										
<5	104	4.8%	221	9.9%	85	11.5%	51	14.7%	36	19.8%
5–10	654	30.0%	667	30.0%	287	39.0%	153	44.1%	82	45.1%
10–20	846	38.8%	832	37.4%	246	33.4%	122	35.2%	56	30.8%
20–50	487	22.3%	419	18.8%	101	13.7%	20	5.8%	8	4.4%
>50	89	4.1%	85	3.8%	15	2.0%	0	0.0%	0	0.0%
unknown	120		76		2		1		0	

## Data Availability

The datasets generated during and analyzed during the current study are available from the corresponding author on reasonable request.
